# Subcutaneous Adipose Tissue–Derived Stem Cell Utility Is Independent of Anatomical Harvest Site

**DOI:** 10.1089/biores.2014.0059

**Published:** 2015-02-01

**Authors:** Mahmood S. Choudhery, Michael Badowski, Angela Muise, John Pierce, David T. Harris

**Affiliations:** ^1^ Tissue Engineering and Regenerative Medicine Laboratory, Advance Research Center of Biomedical Sciences, King Edward Medical University, Lahore, Pakistan.; ^2^ Department of Immunobiology, College of Medicine, The University of Arizona, Tucson, Arizona.; ^3^ Aesthetic Surgery of Tucson, Tucson, Arizona.

**Keywords:** mesenchymal stem cells, regenerative potential, multiple harvest sites

## Abstract

One of the challenges for tissue engineering and regenerative medicine is to obtain suitably large cell numbers for therapy. Mesenchymal stem cells (MSCs) can easily be expanded *in vitro* to obtain large numbers of cells, but this approach may induce cellular senescence. The characteristics of cells are dependent on variables like age, body mass index (BMI), and disease conditions, however, and in the case of adipose tissue–derived stem cells (ASCs), anatomical harvest site is also an important variable that can affect the regenerative potential of isolated cells. We therefore had kept the parameters (age, BMI, disease conditions) constant in this study to specifically assess influence of anatomical sites of individual donors on utility of ASCs. Adipose tissue was obtained from multiple anatomical sites in individual donors, and viability and nucleated cell yield were determined. MSC frequency was enumerated using colony forming unit assay and cells were characterized by flow cytometry. Growth characteristics were determined by long-term population doubling analysis of each sample. Finally, MSCs were induced to undergo adipogenic, osteogenic, and chondrogenic differentiation. To validate the findings, these results were compared with similar single harvest sites from multiple individual patients. The results of the current study indicated that MSCs obtained from multiple harvest sites in a single donor have similar morphology and phenotype. All adipose depots in a single donor exhibited similar MSC yield, viability, frequency, and growth characteristics. Equivalent differentiation capacity into osteocytes, adipocytes, and chondrocytes was also observed. On the basis of results, we conclude that it is acceptable to combine MSCs obtained from various anatomical locations in a single donor to obtain suitably large cell numbers required for therapy, avoiding *in vitro* senescence and lengthy and expensive *in vitro* culturing and expansion steps.

## Introduction

Cell-based therapies are being used as novel therapeutic interventions in tissue engineering and regenerative medicine. Mesenchymal stem cells (MSCs) are found in many adult tissues, expressing CD44, CD73, CD90, and CD105 while lacking expression of typical hematopoietic markers.^1^ MSCs are an attractive cell population for use in autologous therapies due to multipotential differentiation capacity, as well as the potential for tissue repair, hematopoiesis support, and immunomodulation.^2–5^ However, the availability of such cells in sufficient numbers for cell-based therapies has remained a challenge. The requirement for *in vitro* expansion is one of the major disadvantages of MSCs for clinical use. Previous studies have indicated a significant attenuation of the regenerative potential of MSCs with extensive culture,^6,7^ and the safety and efficacy after long-term expansion remains a major concern.

Bone marrow has been a primary MSC source for many years. However, harvest of bone marrow is a highly invasive procedure, and the number, proliferation, and *in vitro* differentiation potential of bone marrow MSCs declines with *in vitro* passaging.^8^ Recently, we and others have succeeded in isolating MSCs from alternative sources such as adipose tissue and cord tissue.^9,10^ Both bone marrow–derived and adipose tissue–derived MSCs share similar morphological, phenotypic, and immunosuppressive properties.^11^ However, easy availability, simple isolation, and greater proliferative potential makes adipose tissue a more practical source compared to bone marrow. Harvesting adipose tissue involves a minimally invasive procedure. Most importantly, fat is widely distributed throughout the body and thus could be obtained from multiple sites, offering an advantageous approach to harvesting large cell numbers from a single procedure without *in vitro* expansion. The present study compared various anatomical locations in several donors in terms of viability, cell yield, frequency, and growth kinetics; and investigated the multilineage differentiation potential of cells isolated from these locations. To extend our findings, we have compared these results with the findings obtained from similar single harvest sites in multiple patients.

The origin of a cell may be important and may influence its biological activities. Previous studies have found variations in cell properties due to differences in age,^12–14^ body mass index (BMI)^15–18^ and disease conditions.^19–21^ Similarly, some studies had been conducted on animals^22^ or only examined differentiation into a single lineage. In our study, however we assessed differentiation into fat, bone, and cartilage, making it unique and the first such study. All donors used in the current study were healthy and comparisons have been performed on fat harvested from multiple depots in the same donors to avoid any variation.

The results of our current study indicate that although there are donor to donor variations, MSCs obtained from multiple harvest sites in a single donor have similar characteristics. Cells from all anatomical sites in donors were comparable in terms of morphology, cell surface phenotype, and differentiation potential. Also, all adipose depots from a single donor exhibited similar yield, viability, MSC frequency, and proliferative potential. Considering the ease of MSC sampling from various anatomical sites along with multilineage differentiation potency, adipose tissue appears to be an ideal source of MSC for cell-based therapies. Furthermore, the results of this study indicate that adipose tissue from various anatomical locations in individual donors can be combined to obtain a suitably large number of MSCs required for clinical use; thus offering an advantageous approach for a single harvest procedure that avoids typical expansion steps in the laboratory.

## Materials and Methods

### Donors and collection of adipose tissue

Human adipose tissue was harvested with a 2.4 mm cannula during a scheduled liposuction procedure. A total of 10 anatomical sites from three donors (designated X, Y, and Z) were studied. The anatomical sites included breast, back, abdomen, deep flank, and axillary of donor X (31-year-old male); Scarpa's fascia, right upper arm, and right flank of donor Y (21-year-old female); and Scarpa's fascia and submental jowl of donor Z (55-year-old female). In addition, we obtained single samples from multiple donors for abdominal *(n*=2), flank *(n*=5), Scarpa's fascia *(n*=17), thigh *(n*=3), and submental jowl *(n*=3) harvest sites. All samples were obtained with written consent from the donors.

### Isolation and expansion of MSCs

Isolation of the MSC-rich stromal vascular fraction was performed by enzymatic digestion as previously described.^9^ Viability of freshly isolated cells was determined by trypan blue exlclusion assay combined with Turks stain to identify the nucleated cells. Cells were plated in 25 cm^2^ culture flasks and maintained at 37°C/5% CO_2_ in humidity. Nonadherent cells were removed 24 h after initial plating and fresh medium was added. The resulting adherent cells were termed adipose tissue–derived mesenchymal stem cells (AT-MSCs). AT-MSCs from all anatomical sites in all donors were processed under the same conditions.

### Flow cytometry

Cultured cells were phenotypically characterized for surface antigen expression by fluorescence-activated cell sorting. Cells at passage 2 were harvested by treatment with 0.05% trypsin/EDTA, resuspended in phosphate buffered saline and counted. Next, 1.0×10^5^ cells were stained for CD3 (BD BioSciences, San Jose, CA), CD14 (BD Immunocytometry Systems, Franklin Lakes, NJ), CD19 (BD BioSciences), CD34 (BD BioSciences), CD44 (BD Pharmingen, Franklin Lakes, NJ), CD45 (BD Pharmingen), CD73 (BD Pharmingen), CD90 (Biolegend, Cambridge, UK) and CD105 (Biolegend) conjugated with Alexa Fluor 700, phycoerythrin (PE), allophycocyanin (APC), PE, APC, fluorescein isothiocyanate, PE, Alexa Fluor 700, and APC, respectively, for 30 min at 4°C. Samples were analyzed with a LSR II flow cytometer (BD Biosciences), and at least 10,000 events were acquired for each sample. Data acquisition and analysis were performed using FACS DIVA software (BD Biosciences).

### Colony forming unit assay

Colony forming unit (CFU-F) assays were performed for each tissue to enumerate the frequency of cells capable of forming colonies. After isolation, cells were plated in 25 cm^2^ culture flasks in serial dilutions in complete medium and incubated at 37°C/5% CO_2_ for 14 days. The resultant colonies were fixed with methanol for 5 min and stained with 0.1% crystal violet for 60 min at room temperature. The flasks were washed with water, and colonies containing more than 30 cells were counted using light microscopy. To score colonies, each sample was counted by at least two independent observers.

### Population doublings and doubling time

MSCs were serially passaged for population doubling analysis as previously described.^9^ The initial confluent cultures were designated as passage 0. Subconfluent cultures were detached with trypsin/EDTA, counted by hemacytometer, and replated at a 1:10 dilution in 25 cm^2^ culture flasks. The final cell number was recorded for each passage until the cells stopped dividing. The cumulative population doublings and doubling time were calculated as described elsewhere.^9,23^

### Adipogenic differentiation

Passage 2 MSCs were seeded in six-well plates in triplicate at a final cell density of 5,000 cells per cm^2^ and propagated in complete medium. Forty-eight hours later, designated as day 0, differentiation was initiated using adipogenic induction medium (AdvanceSTEM adipogenic differentiation medium supplemented with 10% AdvanceSTEM stem cell growth supplement, ThermoScientific, Rockford, IL), as per the manufacturer's instructions. The medium was changed every 3–4 days thereafter, and experiments were terminated after 3 weeks. The differentiated MSCs were fixed with 4% paraformaldehyde (PFA) and stained with oil red O (IHC World, Woodstock, MD) to visualize accumulated cytoplasmic lipid rich vacuoles.^24^ The lipoid bodies were observed under phase contrast microscopy in at least 10 nonoverlapping fields. To quantify staining, oil red O was extracted with isopropanol containing 4% nonidet P-40 detergent and optical density was then measured at 490 nm.^24^

### *In vitro* osteogenic differentiation

Osteogenic differentiation was promoted by treating subconfluent MSC cultures at passage 2 with osteogenic induction medium (AdvanceSTEM osteogenic differentiation medium, supplemented with 10% AdvanceSTEM stem cell growth supplement, ThermoScientific) for 21 days per manufacturer's instructions. Experiments were performed in triplicate. The osteogenic potential was examined for extracellular matrix calcification by the von Kossa's method using a commercially available kit (IHC World). Cultures were treated with silver nitrate for 60 min at room temperature under ultraviolet light, followed by treatment with sodium thiosulphate for 5 min. The cells were counterstained with nuclear fast red and then photographed using phase contrast microscopy. ImageJ software (http://rsbweb.nih.gov/ij/) was used for quantification of mineralized matrix.

### Three-dimensional pellet cultures for chondrogenic differentiation

Chondrogenesis was induced in micromass pellet cultures as described.25 Micromass-pellet cultures were prepared from 1.0–2.5×10^5^ MSCs (passage 2) in 15-mL polypropylene tubes that were centrifuged at 150 *g* for 10 min in complete medium. Cell pellets were incubated with induction medium (AdvanceSTEM chondrogenic differentiation medium, ThermoScientific) for 3 weeks. Each pellet was parafinized after dehydration and cut into thin sections (4–5 μm). The sections were analyzed for chondrogenic differentiation using a commercially available Alcian blue kit (IHC World, Woodstock). For staining, sections were fixed with 4% PFA and washed with distilled water followed by treatment with Alcian blue for 20 min. Alcian blue uptake was analyzed using a colorimetric assay as described.^26^ After 21 days, the micromass cultures were fixed with methanol and whole mount stained with Alcian blue. Alcian blue was extracted with 6M guanidine HCl and absorbance was read at 620 nm. All experiments were performed in triplicate.

### Quantitative RT-PCR

To evaluate differentiation, the expression of lineage specific genes related to each tissue was assayed at the mRNA level by RT-PCR. Total RNA was isolated from differentiated and undifferentiated cells using Trizol reagent (Invitrogen, Grand Island, NY) and an Rneasy Mini Kit (Qiagen, Valencia, CA), according to the protocol recommended by manufacturers for cultured cells. RNA concentration was determined using a ND-1000 spectrophotometer (NanoDrop Technologies, Wilmington, DE), cDNA was synthesized using oligo dt primers (10 μM), and reverse transcriptase enzyme performed with the SuperScript III First Stand synthesis system (Invitrogen). To minimize PCR reaction variations, all samples from individual donors were transcribed simultaneously.

Quantitative RT-PCR was performed using an ABI PRISM 7300 sequence detection system. The final reaction contained template cDNA, iTaq SYBR Green supermix with ROX (Bio-Rad, Hercules, CA) and gene-specific primers as shown in Table 1.^9^ The following PCR conditions were used: 50°C for 2 min and 95°C for 10 min followed by 40 cycles for 30 sec at 95°C, 45 sec at 60°C and 72°C for 30 sec. Beta actin was used as an internal control. The CT (cycle threshold) values of beta actin and specific genes were acquired after polymerase chain reaction. The normalized fold expression was obtained by the 2^-ΔΔCT^ method.^27^ The results are expressed as the normalized fold expression for each gene.

**Table 1.** Gene-Specific Primers

**Gene**	**Primer sequences (5′-3′)**
Collagen type 2	GGCAATAGCAGGTTCACGTACA (F)
	CGATAACAGTCTTGCCCCACTT (R)
Osteocalcin	GGCAGCGAGGTAGTGAAGAG (F)
	CTGGAGAGGAGCAGAACTGG (R)
Lipoprotein lipase	GTCCGTGGCTACCTGTCATT (F)
	TGTCCCACCAGTTTGGTGTA (R)
Aggrecan	TCAACAACAATGCCCAAGAC (F)
	AGCGACAAGAAGAGGACACC (R)
Alkaline phosphatase	GACCCTTGACCCCCACAAT (F)
	GCTCGTACTGCATGTCCCCT (R)
PPAR-γ	AAGACCACTCCCACTCCTTTG (F)
	GTCAGCGGACTCTGGATTCA (R)
Beta actin	AGAGCTACGAGCTGCCTGAC (F)
	AGTACTTGCGCTCAGGAGGA (R)

F, forward; PPAR-γ, proliferator-activated receptor gamma; R, reverse.

### Statistical analysis

The Graphpad Prism 5 Software was used for statistical analysis. One-way ANOVA was used when three or more groups within one variable were compared. To analyze two groups the unpaired *t*-test was used. The data are expressed as mean±standard error of the mean. Values of *p*<0.05 were considered significant.

## Results

### Morphology and phenotype

**FIG. 1.**
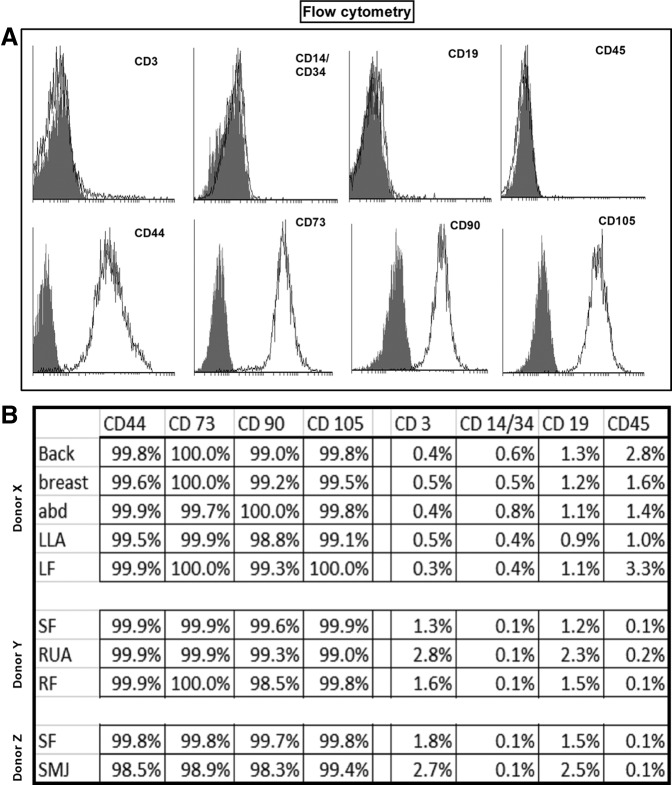
Phenotypic characterization of adipose tissue–derived mesenchymal stem cells (AT-MSC) from different anatomical harvest sites. ABD, abdomen; LF, left deep flank; LLA, left lateral axillary; RF, right flank; RUA, right upper arm; SF, Scarpa's fascia; SMJ, submental jowl. Flow cytometric analysis of AT-MSCs indicating positive expression of CD44, CD73, CD90, and CD105, while lacking expression of the hematopoietic markers CD3, CD14, CD19, CD34, and CD45. **(A)** Representative fluorescence-activated cell sorting graphics; **(B)** analysis of cell surface antigen expression.

Cells from all harvest sites in multi-site and single-site donors showed similar fibroblastic morphology (data not shown). MSCs from all harvest sites were negative for hematopoietic markers CD3, CD14, CD19, CD34, and CD45 and positive for expression of the mesenchymal markers CD44, CD73, CD90, and CD105. Figure 1 shows representative flow cytometric data for each marker with the percentage expression of each marker shown. These MSC phenotype results were in agreement with previous reports.^28,29^

### Effect of harvest site on AT-MSC viability, nucleated cell yield and CFU-F frequency

**FIG. 2.**
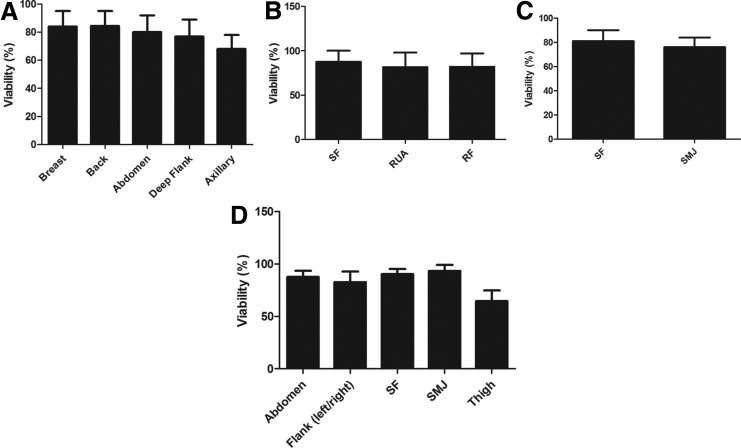
Viability of cells isolated from each anatomical locations in the individual donors. Percentage viability was compared for AT-MSCs obtained from multiple anatomical sites in each donor **(A–C)**. **(D)** Viability of AT-MSC isolated from five anatomical locations in multiple independent single-site donors.

**FIG. 3.**
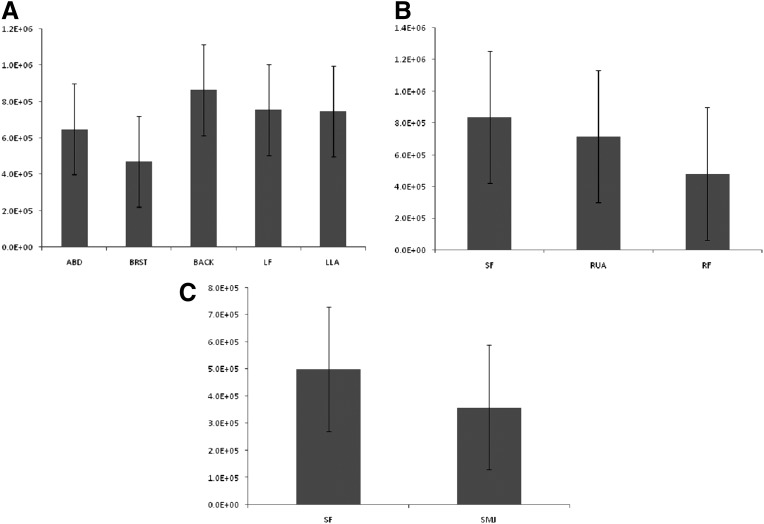
Nucleated cell yield based on harvest site. The yield of nucleated cells in the stromal vascular fraction was determined for the indicated harvest site in each donor **(A**, **B**, and **C)**.

**FIG. 4.**
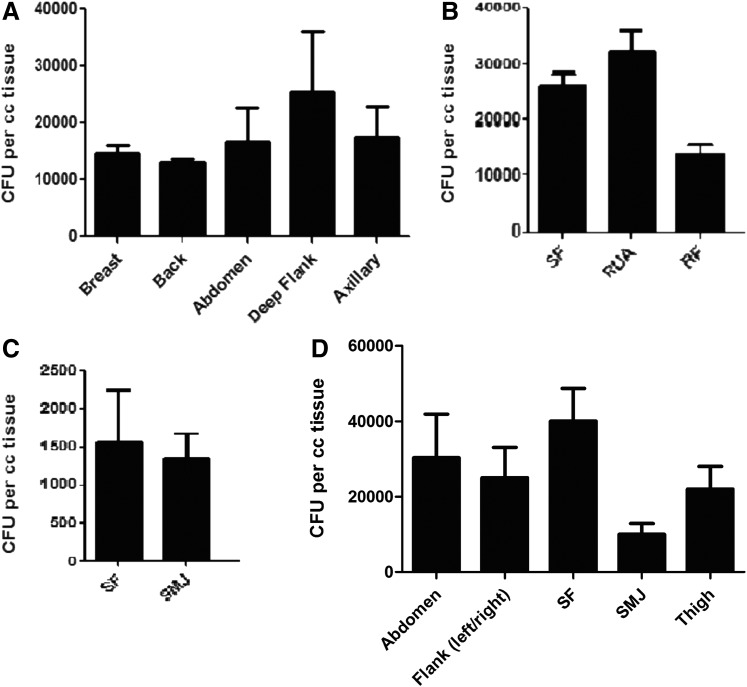
MSC frequency based on harvest site. Colony forming unit (CFU-F) assays were enumerated on day 14 to determine MSC frequency as described in “Methods.” The effect of anatomical location is shown for donor X **(A)**, Y **(B)**, and Z **(C)**. In addition, CFU-Fs produced by five unique harvest sites from multiple single-site donors are shown in **(D)**.

All samples were analyzed using equal amounts of adipose tissue. The percentage viability was equivalent for all anatomical locations in each multi-site donor as shown in Fig. 2A (donor X), 2B (donor Y), and 2C (donor Z). Figure 2D shows percentage viability in different anatomical sites of the individual single-site donors. Similarly, the yield of nucleated cells per cc of adipose tissue was determined and the results showed no significant difference between sites (Fig. 3A–C). Although there were significant variations in CFUs between donors, we observed no significant difference in the CFU frequency of MSCs harvested from any of the various sites in an individual multi-site donor (Fig. 4A–C). When CFU numbers obtained from corresponding single-site patients were considered, no significant difference was observed except for the submental jowl harvest site (Fig. 4D), that produced significantly fewer CFUs.

### Effect of anatomical site on MSC growth characteristics

**FIG. 5.**
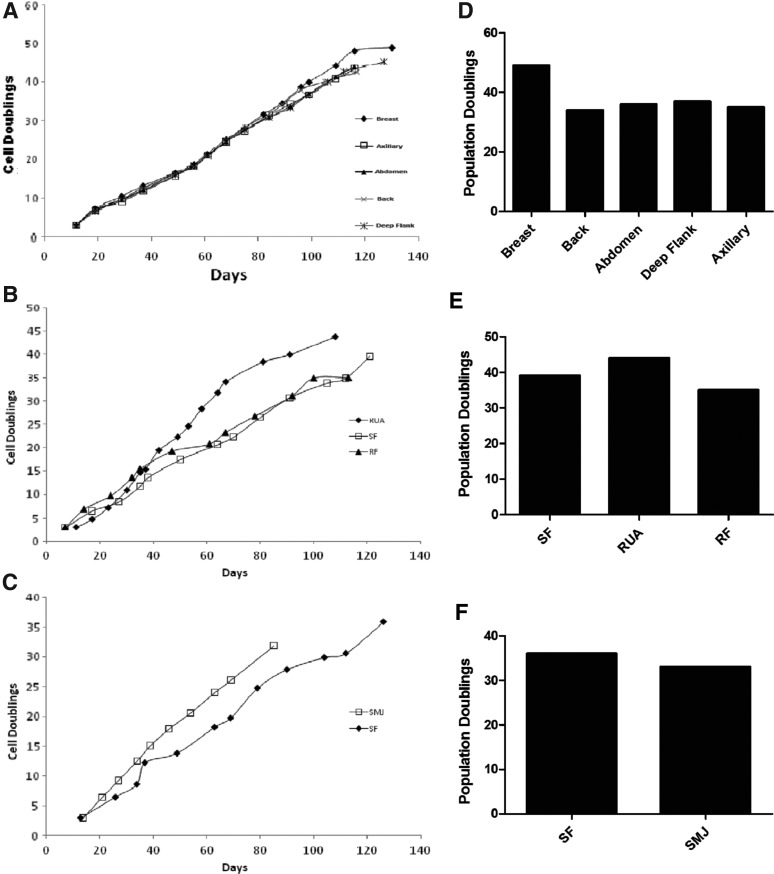
Effect of anatomical site on the growth characteristics of AT-MSCs. **(A–C)** The growth curves for each anatomical harvest site in individual donors and the number of maximum population doublings for these anatomical sites **(D–F)** are shown.

**FIG. 6.**
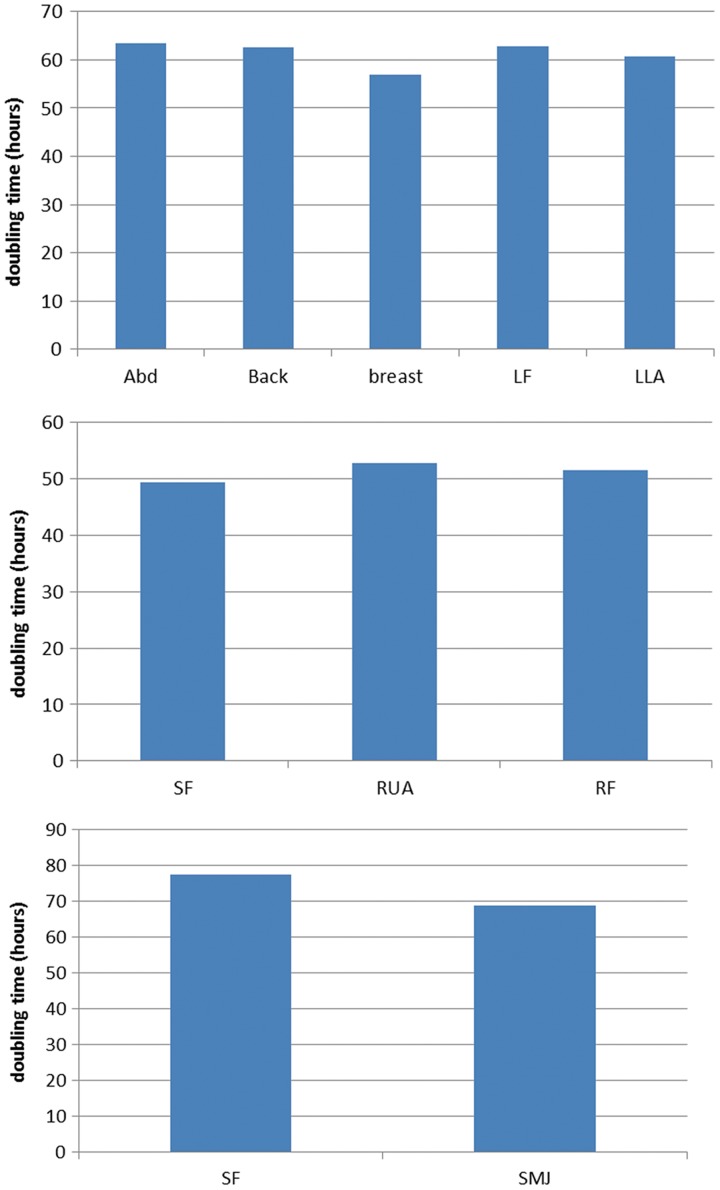
Effects of AT-MSC harvest site on growth kinetics. MSCs isolated from each anatomical site in each donor were serially passaged, and initial and final cell numbers were recorded to measure population doubling times as described. Donor X (*top*), donor Y (*middle*), and donor Z (*bottom*).

To investigate the effect of anatomical site on MSC growth characteristics, we determined the maximum population doublings (Fig. 5A–C). Cells from different sites in the same donor showed similar growth curves. Cells from all anatomical sites expanded rapidly and there was no significant difference in maximum population doublings (Fig. 5D–F). The maximum number of population doublings for MSCs isolated from each donor X, Y, and Z was 48.20±2.746, 39.33±2.603, and 34.50±1.500, respectively. Similarly, doubling times were also similar for MSCs isolated from different anatomical locations in the same donor (Fig. 6).

### MSCs harvested from different anatomical locations have equivalent differentiation capacity

**FIG. 7.**
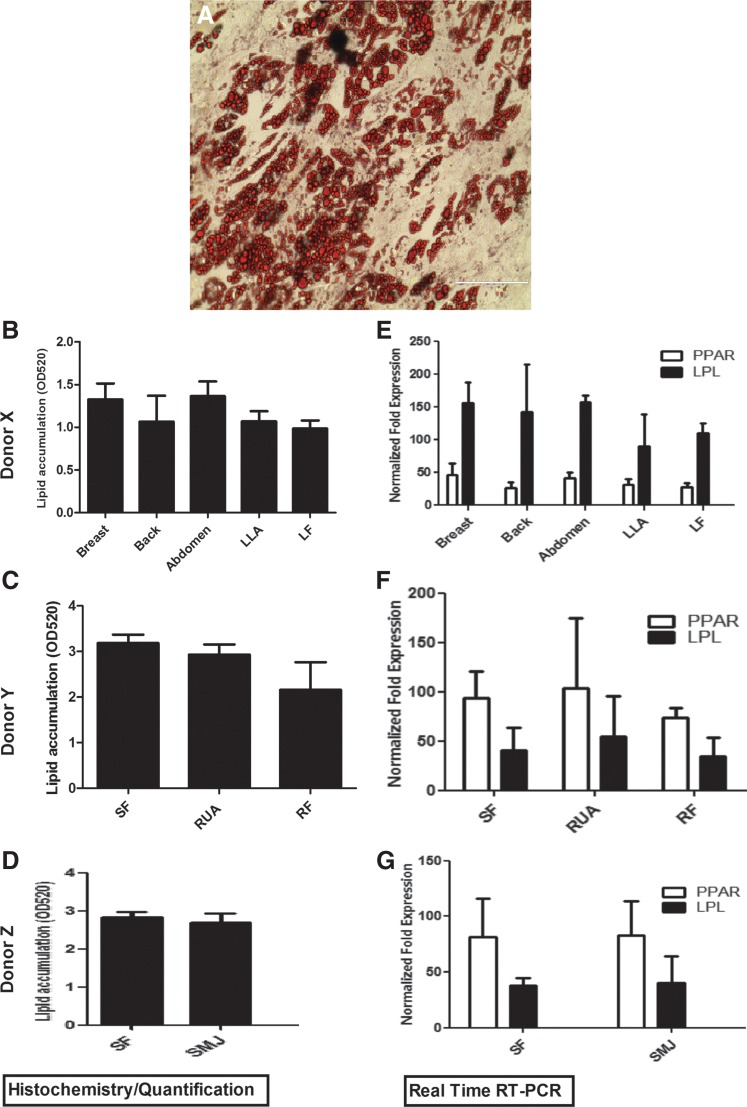
Assessment of adipogenic induction based on harvest site. MSCs from each anatomical location in donors X, Y, and Z were induced for 21 days and differentiation potential was assessed as described in “Methods.” **(A)** Representative slide showing oil red O staining. **(B–D)** Analysis of oil red O uptake among each anatomical site in donors X, Y, and Z. **(E–G)** Analysis of lineage-specific mRNA levels of LPL and PPAR-γ. LPL, lipoprotein lipase; PPAR-γ, peroxisome proliferator-activated-receptor-gamma.

Lipid droplets in the induced cultures showed positive staining for adipogenesis as shown in Fig. 7A. Quantification of oil red O uptake^24^ indicated no significant difference between the various anatomical locations of each individual donor site (Fig. 7A–C). Differentiation was confirmed by quantitative PCR analysis of mRNA levels of the adipogenic markers, peroxisome proliferator-activated receptor gamma, and lipoprotein lipase (Fig. 7E–G).

**FIG. 8.**
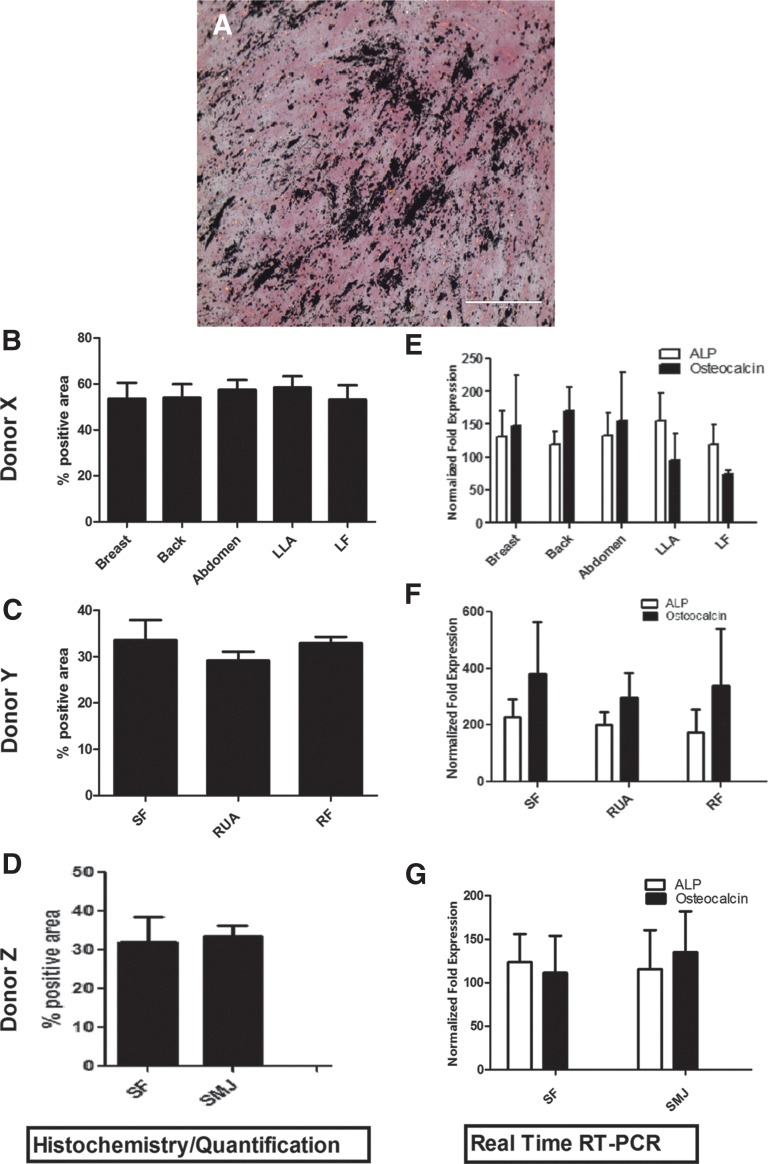
Analysis of harvest site effects on osteogenic induction. **(A)** Representative slide showing von Kossa staining of AT-MSCs isolated from each anatomical site in each individual donor. **(B–D)** Matrix mineralization in each induced sample as analyzed using ImageJ software (**B**, donor X; **C**, donor Y; **D**, donor Z). **(E–G)** Quantitative analysis of steogenic-associated gene expression with real time RT-PCR. Values are expressed as the mean±standard error of the mean (SEM).

After 3 weeks in culture, Von Kossa staining was performed on induced MSCs to identify osteogenic differentiation by extracellular matrix deposition specifically secreted by osteoblasts, as shown in Fig. 8A. Von Kossa staining revealed positive and equivalent staining of extracellular matrix formation in induced MSCs harvested from each of the various anatomical locations [Fig. 8B (donor X), Fig. 8C (donor Y), and Fig. 8D (donor Z)]. Osteogenic induction was further evaluated by real time RT-PCR analysis of lineage-specific expression of alkaline phosphatase and osteocalcin. A significant up-regulation of the genes was observed in the induced MSC cultures, with similar expression profiles in all MSC harvest sites (Fig. 8E–G).

**FIG. 9.**
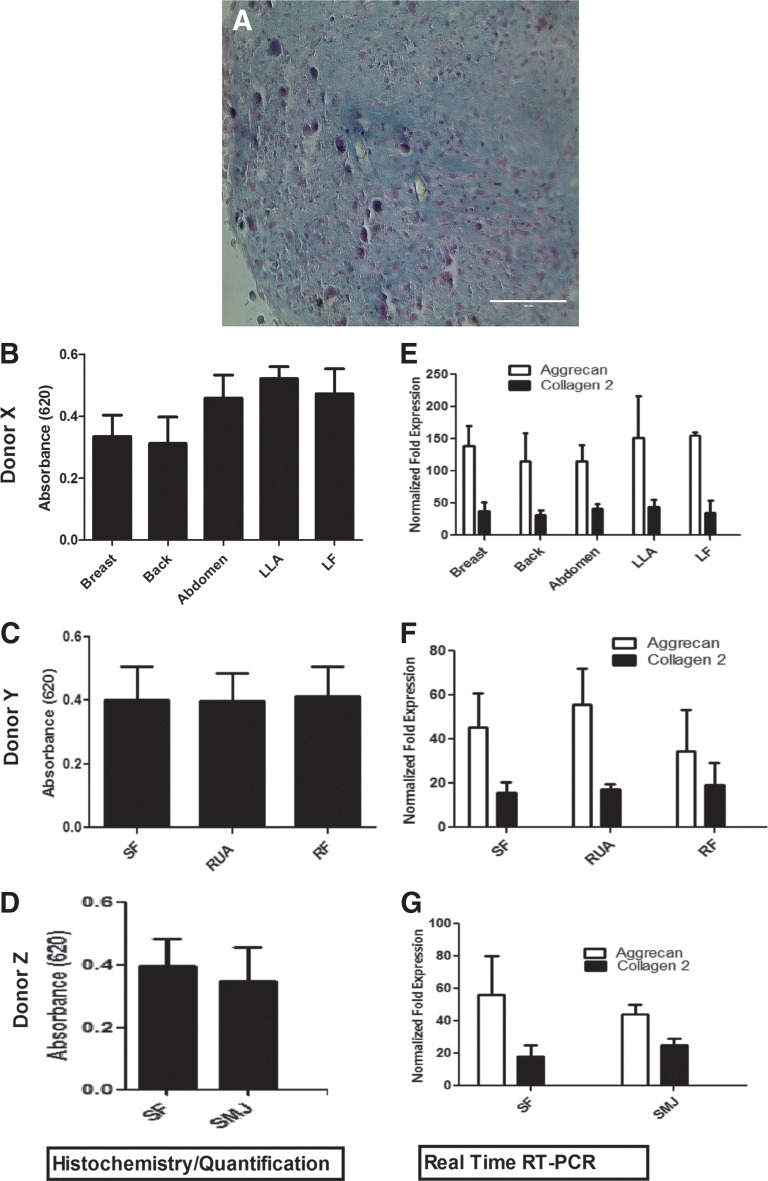
Chondrogenic potential of MSCs isolated from various anatomical locations. AT-MSCs obtained from each anatomical site in each donor were subjected to chondrogenic induction as described. **(A)** Alcian blue staining of glycosaminoglycans and mucopolysaccharides within the extracellular matrix of induced AT-MSC cultures. **(B)** Quantitation of Alcian blue dye uptake in harvest sites of donor X; **(C)** donor Y; and **(D)** donor Z. **(E–G)** Quantitative RT-PCR analysis of aggrecan and collagen type 2 mRNA expression based on harvest site. The values are expressed as mean±SEM.

Chondrogenic differentiation of AT-MSCs was induced by micromass pellet cultures. Uptake of Alcian blue staining^24^ (Fig. 9A) revealed no quantitative differences (Fig. 9B–D) with MSCs obtained from each anatomical location in an individual donor. RT-PCR analysis of collagen type 2 and aggrecan genes revealed no significant differences in the chondrogenic differentiation potential of MSCs derived from each anatomical location in an individual donor (Fig. 9E–G).

## Discussion

MSCs proliferate rapidly *in vitro* and readily differentiate into cells of mesenchymal and nonmesenchymal origin. Generally, MSCs have been obtained from bone marrow, but low cell yields and an invasive harvest procedure make it a less desirable cell source. AT-MSCs can be easily harvested under local anesthesia in large numbers and induced to differentiate into multiple lineages. Early preclinical and clinical reports indicate that MSC therapies are safe and effective.^34^ Recent achievements with adoptively transferred MSCs have made these cells a preferred cell type for tissue engineering. However, one major disadvantage of most MSC sources is the low numbers found in most tissues, which usually requires expansion before use. Unfortunately, the fundamental properties of the MSCs may change during long-term culture,^35^ raising questions about safety and efficacy. Attenuation of MSC differentiation potential during *ex vivo* expansion due to senescence has been reported^36^ and could be a major obstacle in the utilization of MSCs for tissue engineering applications. Our findings reported herein have demonstrated that adipose-derived MSCs obtained from various anatomical locations in the same donor could be combined to obtain suitably large cell numbers needed for therapy without extensive expansion, avoiding *in vitro* senescence and lengthy and expensive *ex vivo* cultures.

In this study we have investigated whether different anatomical sites in an individual donor influence the nature, growth characteristics, and differentiation potential of their AT-MSC. Despite abundant information on the regenerative potential of AT-MSCs, rather little information is known regarding the effects of harvest site on MSC function. The biological origin of a cell can determine its biological activity and clinical applications thus might depend on cell niche.^37^ Functional and biological properties of MSCs derived either from different “sources,”^38^ and “anatomical sites”^22,39–44^ have been reported. In our study, we assessed the ability of AT-MSC harvested from different anatomical sites in the same (or different) donors to differentiate into fat, bone, and cartilage, making the study the first study of its kind. The data presented herein was obtained from 3 multi-site donors (with adipose tissue harvested from 5, 3, and 2 independent locations in the same donor) and up to 17 single-site donors (with adipose tissue harvested from one site), for comparison.

We successfully isolated AT-MSCs from each of the anatomical sites under study and found no significant differences in cell viability based on different anatomical locations, in agreement with other investigators.^44^ Similarly, the yield of nucleated cells per cc of fat was similar for each adipose tissue harvested. Previously, Jurgen et al.^30^ found differences in cell yield between the abdomen and the thigh/hip region. However, this study compared harvests from different donors rather than from the same donor. We also noticed no differences in CFUs obtained from each of the anatomical locations in an individual donor. To validate our findings, we compared these results with data obtained from multiple donors with single harvest sites, with similar results being obtained. Using International Society for Cellular Therapy definitions,^1^ our results have indicated that cells isolated from each adipose tissue site were “true” MSC based on morphology, phenotype, and function. AT-MSCs can be harvested from each tissue site, expanded *in vitro*, and differentiated into cells of multiple lineages.^9^ Microscopic observation revealed a spindle-shaped morphology of MSCs harvested from each different location, as previously reported for bone marrow-derived MSCs and AT-MSCs,^45,46^ with uniform expression of MSC markers (CD44, CD73, CD90, CD105), absence of hematopoietic markers (CD3, CD14, CD19, CD34, CD45), and ability to differentiate into adipose, bone, cartilage, and neural lineages, consistent with results reported by others.^47,48^

MSCs from each anatomical location were highly proliferative and found in large numbers. The number of population doublings was similar for each anatomical site in each of the individual donors, in conflict with some previously published reports on the replicative potential of AT-MSCs isolated from different locations. Van Harmelen et al.^14^ reported that subcutaneous ASCs proliferated faster than those from the omental region, while Roncari et al.^49^ and Petterrson et al.^50^ found no such differences. However, these observations might be explained by variable methodologies and by harvesting adipose cells from different donors instead of collecting adipose tissue from different locations in the same donor. Jurgen et al.^30^ found similar growth kinetics when comparing adipose stem cells from the abdomen and hip/thigh regions. We also observed some variation in doubling time between donors but not between anatomical sites in the same donor.

Upon lineage-directed differentiation, AT-MSCs from each site displayed morphology and phenotype of cells of the adipogenic, condrogenic, and osteogenic lineages. In addition, up-regulation of the lineage-specific genes as measured with real time RT-PCR further confirmed the equivalent differentiation potential of multiple harvest sites. Previously, in a report specifically comparing the adipogenic potential of abdominal subcutaneous and omental ASCs obtained from obese and control subjects, no differences were observed.^50,51^ In terms of multi-differentiation very little has been reported regarding adipose harvest site. However, Tckkonia and colleaques^52^ reported that subcutaneous adipose cells differentiate more extensively than omental AT-MSCs. However, when we analyzed the effect of harvest site within the same healthy donor we found no significant difference based on morphology or lineage-specific gene expression in terms of adipogenesis, osteogenesis, or chondrogenesis differentiation.

## Conclusions

Adipose tissue as a source of MSCs for clinical translation is attractive because it can be easily obtained and stored (cryogenically banked) for later use. Furthermore, fat is widely distributed throughout the body and thus could be obtained easily from multiple sites. It was therefore important to determine the potential capabilities of adipose tissue–derived MSCs obtained from various anatomical locations. In this comparative study, we demonstrated that MSCs obtained from various anatomical locations in an individual donor were comparable in terms of phenotype and differentiation into cells of adipogenic, osteogenic, and chondrogenic lineages. Adipose tissue harvested from each site in each donor displayed similar yields, viability, and CFU frequency. Further, we observed similar growth kinetics of MSCs derived from each anatomical site. These results were confirmed with data from multiple (*n*=30) donors in which an individual, single harvest site was analyzed. Therefore, AT-MSC from different harvest sites in a donor could be combined as novel alternative sources of MSCs for tissue engineering and regenerative medicine. This study provides important impetus for the use of MSCs collected from different anatomical locations during a single surgical procedure for regenerative medicine and tissue engineering applications, thus avoiding lengthy and expensive *in vitro* culture and expansion steps.

## Abbreviations Used

APC: allophycocyanin
ASC: adipose tissue–derived stem cell
AT-MSC: adipose tissue–derived mesenchymal stem cell
BMI: body mass index
CFU-F: colony forming unit
CT: cycle threshold
MSC: mesenchymal stem cell
PE: phycoerythrin
PFA: paraformaldehyde
